# Inflammation markers are associated with small extracellular vesicle protein signatures in cows with *Mycoplasma bovis*

**DOI:** 10.3389/fvets.2026.1787282

**Published:** 2026-05-15

**Authors:** Mallory A. Ross, Evelyne Maes, Alice V. R. Lake, Joel Pratt, Charles Hefer, Ancy Thomas, Amy Burroughs, Douglas Begg, Murray David Mitchell, John R. Roche, Axel Heiser

**Affiliations:** 1 Bioeconomy Science Institute, AgResearch Group, Hopkirk Research Institute, Palmerston North, New Zealand; 2 Bioeconomy Science Institute, AgResearch Group, Lincoln, New Zealand; 3 OSPRI New Zealand Ltd, Wellington, New Zealand; 4 Ministry for Primary Industries, Animal Health Laboratory, Wellington, New Zealand; 5 Institute of Health and Biomedical Innovation, Queensland University of Technology, Brisbane, QLD, Australia; 6 School of Biological Science, University of Auckland, Auckland, New Zealand

**Keywords:** biomarker, cattle, dairy cows, diagnostics, exosomes, proteomics

## Abstract

**Introduction:**

*Mycoplasma bovis (M. bovis)* manifests as diverse clinical pathologies in cattle that significantly impacts cow health and productivity worldwide. Current diagnostic methods rely on active infection to detect antibodies and bacterial DNA, however, these methods could be improved for subclinical infections. This is of particular importance in long-term surveillance programs in countries that have decided to eradicate *M. bovis*, such as New Zealand.

**Methods:**

This study investigated small extracellular vesicles (sEV) from serum samples of *M. bovis* infected dairy cows to identify protein cargo that may be used as potential diagnostic markers during subclinical infection to complement current testing methods. Small EV were isolated from serum of dairy cows positive for *M. bovis* infection (*n* = 45) and their protein cargo compared with those from serum sEV from non-infected dairy cows (*n* = 49). Proteins were identified using liquid chromatography–ion mobility–tandem mass spectrometry in pooled samples (*n* = 10/group), resulting in 695 non-redundant top proteins identified across all samples.

**Results:**

Differential protein abundance analysis indicated 90 proteins significantly (*q*-value < 0.05) different in sEV of infected animals compared with controls. Proteins associated with inflammation and the complement system as well as proteins involved in the oxidative stress pathway, were in greater abundance in sEV from *M. bovis* positive animals compared with *M. bovis* negative animals. Several histone proteins and antimicrobial peptides exhibited lower abundance in sEV from *M. bovis* positive compared with negative cows.

**Conclusion:**

Although this study has not identified a protein candidate specific enough for use as a single diagnostic marker, our results indicate a shift towards a diseased state in *M. bovis* infected dairy cows and provide valuable insight into sEV biology during *M. bovis* infection.

## 1 Introduction

*Mycoplasma bovis* (*M. bovis*) is a globally significant pathogen of cattle, causing significant economic burden due to its impact on cattle health and productivity. *M. bovis* is the primary cause of bovine mycoplasmosis, which manifests in diverse clinical conditions, including mastitis (1), bronchopneumonia (2), arthritis (3), uterine inflammation, and infertility (4). Notably, it is a leading cause of bovine respiratory disease (5), posing a significant burden on the global beef and dairy industries.

The economic impact of *M. bovis* infections is substantial. In the United States alone, the annual costs associated with bovine respiratory disease are estimated at over USD$55 million, with a third or more likely due to *M. bovis* infection (5, 6). This excludes annual costs associated with morbidity and mortality of cattle losses due to other health conditions; costs associated with reduced fertility, premature culling, and decreased production are estimated in the hundreds of millions (7). The challenge of managing *M. bovis* infections is exacerbated by antibiotic resistance to commonly used beta-lactam antibiotics and increasing antibiotic resistance to non-beta-lactam antibiotics, as well as the limited efficacy of available vaccines (8, 9). Consequently, most management strategies rely on infection prevention and antibiotic treatment. In some cases, bacterin or autogenous vaccines are also used, despite limited evidence for their clinical benefits (10–12).

In contrast to the policies of other countries, New Zealand adopted a unique approach to *M. bovis* after its first detection in 2017 (13). The government, in partnership with industry, launched an eradication campaign that included compulsory depopulation of affected herds, strict movement controls, and the implementation of novel diagnostic and surveillance techniques to support disease detection and management. Without such measures, the economic impact of untreated *M. bovis* in New Zealand was projected to reach NZD$1.3 billion over 10 years. The eradication program is proving successful at mitigating economic losses and preventing long-term animal welfare issues associated with widespread outbreaks.

Recent advances in molecular and proteomic tools have highlighted the importance of novel diagnostic approaches for long-term surveillance of *M. bovis* infection where monitoring will continue for several years post-eradication. Conventional methods, including bacterial isolation, polymerase chain reaction (PCR), and serological tests, such as enzyme-linked immunosorbent assay (ELISA) remain the routine testing methods, but the results vary depending on sample type and stage of infection (14). Traditional bacterial isolation is the gold standard for confirming *M. bovis* infection; however, it is slow, has demanding technical requirements, and can lack sensitivity in chronic and subclinical infections (15, 16). Molecular methods, such as PCR, offer improved sensitivity and rapid results but may detect non-viable organisms and demonstrate variable performance depending on sample type and stage of infection (17, 18). Serological tests, such as ELISA, are useful for herd-level surveillance but have reduced sensitivity and specificity in diagnosing early or subclinical infections (19, 20). The limitations in these current methods of detection highlight the need for improved diagnostic approaches; assays based on *M. bovis*-specific antigens may improve sensitivity and specificity across different disease stages, complementing current testing for long-term surveillance.

Small extracellular vesicles (sEV), including exosomes, are nanoscale vesicles (~30 to 200 nm) released by all cell types into biological fluids, making them a source of non-invasive “liquid biopsies” (21). Small EV are produced as part of an endosomal pathway and expelled into the extracellular space and, thereby, carry protein, RNA, and DNA representative of the metabolic state of their tissue of origin (22). In dairy cows, sEV have been identified in milk, blood (plasma or serum), saliva, and urine, indicating their role in physiological and pathological processes (23). Serum EV proteins are important for cellular communication in dairy cows and have roles in modulating the immune system in health and disease (24). During *M. bovis* infection, sEV serve as critical mediators in pathogen–host communication and effectors of the immune response. For example, sEV derived from infected lung and mammary epithelial cells *in vitro* effectively sensitize and activate recipient macrophages, triggering a significant production of pro-inflammatory cytokines (25, 26). These unique characteristics of sEV have driven a growing interest in their use as diagnostic tools for monitoring disease progression. Their importance in the context of bovine diseases, including *M. bovis* infections, could provide a transformative approach to early and precise diagnosis, improving disease management and outcomes.

In this study, we investigated whether protein signatures of *M. bovis* can be identified in serum sEV of infected dairy cows, which could be used for the development of surveillance diagnostics.

## 2 Materials and methods

### 2.1 Animals

Serum samples were collected either under the Biosecurity Act 1993 New Zealand or animal ethics approvals from Massey University or AgResearch New Zealand in accordance with the New Zealand Animal Welfare Act (27). The samples taken under the Biosecurity Act 1993 were collected as part of the *M. bovis* eradication program, with the permission of the owner of the cattle to use the samples beyond the scope of the eradication for research purposes. Negative samples were collected by AgResearch NZ under ethics approval AE Application 15,028 approved by the AgResearch Animal Ethics Committee. The additional samples were collected by Massey University using ethics approval number MUAEC Protocol 20/52 approved by the Massey University Animal Ethics Committee.

Dairy cows of mixed age (>2yo) and breed (Holstein-Friesian × Jersey and Holstein-Friesian) were selected based on their *Mycoplasma bovis* infection status from participating farms across New Zealand. Sample source locations and additional information on the health status of the animals are confidential due to privacy, and we have provided as much context and detail as permissible. Cows were from three commercial dairy farms using standard seasonal calving management in New Zealand. All animals were grazing dairy cows and due to the seasonal nature of calving in New Zealand, we can infer from the sampling dates that animals were outside the transition (calving) period and likely lactating and pregnant, serum sample pooling (Section 2.4) reduced variation across infection status groups. All dairy cows were considered clinically healthy based on veterinary observation that there was an absence of clinical pathology at the time of blood collection; only one serum sample was used from each animal. Sample collection from *M. bovis* negative (*n* = 49) animals occurred in May 2020 from one North Island farm (*n* = 24) and one South Island farm (*n* = 25). Sample collection from *M. bovis* positive (*n* = 45) animals occurred in December 2020 (*n* = 37), January 2021 (*n* = 2), and March 2021 (*n* = 6), from different herds all from a single farm in the South Island of New Zealand.

### 2.2 Sample collection and processing

Blood (8 mL) was collected in serum collection blood tubes (Vacutainer, BD), inverted 8 times, and left at room temperature for sufficient time to allow for the activation of the clotting process. Blood was centrifuged (1,200×*g* for 10 min), and 5 mL of serum was aliquoted and stored at −20 °C until processing for sEV isolation.

### 2.3 Confirmation of infection status

Infection status was confirmed by screening serum samples using the ID Screen® *Mycoplasma bovis* Indirect ELISA Kit (IDvet MBOVISS-10P), according to manufacturer’s instructions. The relative amount of antibodies in the samples was calculated as [sample optical density (OD) − negative control OD]/[positive control OD − negative control OD] × 100 (S/P%). The infection status cutoff for a positive sample was set to S/P% ≥ 60% (New Zealand Ministry for Primary Industries). The average S/P% was 7% ± 3.5% (mean ± SD) for the *M. bovis* negative samples and 101% ± 18.0% (mean ± SD) for *M. bovis* positive serum samples.

### 2.4 Small EV isolation and characterization

Serum samples were defrosted at room temperature and clarified by centrifugation for 10 min at 1,500×*g*. The supernatant was further clarified by centrifugation for 10 min at 10,000×*g*. Serum samples were pooled according to infection status (negative or positive), farm location, and breed (Friesian/Jersey cross and Friesian; Supplementary Table 1). Samples were made up to 10 mL of total serum and topped up to 10 mL using 1 × PBS if there was not enough serum. This resulted in *n* = 10 samples in each group (*M. bovis* negative and positive).

Small EVs were isolated from pooled serum samples by size exclusion chromatography (SEC) using IZON qEV10 35 nm Gen 2 columns on an automatic fraction collector (AFC) (IZON Science) to collect 5 mL elution fractions into protein LoBind 5 mL tubes (Eppendorf). Three sEV-containing fractions were pooled (Fractions 8–10) as per manufacturer’s recommendations and the presence of sEV-associated proteins (Section 2.4.3). Pooled fractions were concentrated using Amicon Ultra–15 Centrifugal Filter Units (Ultracel–100 membrane) (Merck–Millipore) by centrifuging at 4,000×*g* for 10 min at 25 °C or until the retentate reached approximately 250 μL. The retentate was transferred to LoBind tubes (Eppendorf), and 10% of the total retentate volume was removed. From the fraction removed, 10 μL was allocated for analysis of sEV concentration using nanoparticle tracking analysis (NTA), with the remaining volume fixed for imaging using transmission electron microscopy (TEM). The remaining 90% of the retentate was lysed using a protein lysis buffer and stored at −20 °C for subsequent analysis or protein extraction.

#### 2.4.1 Nanoparticle tracking analysis (NTA)

Concentrated sEV from pooled serum isolation was diluted (1:100–1:800 depending on sample) in 1 × PBS for NTA using a NanoSight NS300 instrument (Malvern Panalytical, UK). Run settings included a camera level of 13 and pump speed of 300. Three videos per sample were captured for analysis with a modal size and concentration of sEV across all three videos reported. Analysis settings included a detection threshold of 10. Water (1 mL; Milli-Q, Merck Millipore) was used to clean the line between samples, and 2 × water (1 mL) and 1 × 5% ethanol (500 μL) were infused after each batch of samples on a given day.

#### 2.4.2 Transmission Electron microscopy (TEM)

Concentrated sEV isolations were fixed in 2 × volume of 3% (v/v) glutaraldehyde/0.1 M sodium cacodylate buffer (pH7.2) for ≥24 h. Square mesh (200) 3 mm (Agar Scientific) Formvar/Carbon coated copper grids (coated in-house by Manawatū Microscopy and Imaging Centre) were inverted into 10 μL of fixed sample for 4.5 min at room temperature. The grids were washed once with Milli-Q water, and the sample was then negatively stained by transferring the grid to a 10 μL droplet of 4% (w/v) uranyl acetate solution (aqueous). The grids were incubated for 4.5 min, and excess liquid was blotted using filter paper. The images were then captured using a FEI Tecnai G2 Spirit BioTWIN Transmission Electron Microscope (Field Electron and Ion Company, Thermo Fisher Scientific).

#### 2.4.3 Western blot

Lysates of sEV were separated by SDS-PAGE using Bis–Tris Gels (15–well 1.5 mm 4–12%); (Thermo Fisher Scientific) and blotted onto a PVDF membrane using the NuPAGE system and reagents (Invitrogen™, Thermo Fisher Scientific). Membranes were blocked in 5% [w/v] BSA/TBS-T (0.1%). Post-blocking, membranes were separated into individually labeled 50 mL conical tubes and washed twice with 5 mL TBS-T (0.1%) and immunoblotted using anti-protein rabbit primary antibodies against CD9, heat shock protein 70 (Exo–Ab–Kit–1, System Biosciences), syntenin–1 (Ab19903, Abcam), or calnexin (Ab22595, Abcam), all used diluted at 1/1,000 in blocking solution. Membranes were continually rotated using a Rotator Multi-mix with adjustable adaptors (Avantor, VWR International) in the primary antibody for 24 h at 4 °C. Membranes were then washed five times with 5 mL of TBS-T and transferred to a fresh 50 mL conical tube containing secondary antibody diluted 1/10,000 in blocking solution. Membranes were constantly rotated in secondary antibody for an hour at room temperature. Finally, membranes were washed five times for 2 min each wash in TBS-T (0.1%).

Proteins were visualized with Clarity™ ECL Western Blotting Substrate (Bio-Rad) using a 1:1 ratio of luminol–enhancer solution and peroxide solution in the dark. Membranes were imaged using a ChemiDoc MP Imaging System (Bio-Rad). Protein ladders were imaged using automatic optimal colorimetric settings. Protein bands were imaged using chemiluminescence settings. All ladder and protein band images were merged/processed using ImageLab (Bio-Rad).

#### 2.4.4 ExoCheck™ exosome antibody Array

ExoCheck: Exosome antibody array (System Bioscience) was used to detect EV markers CD63, CD81, ALIX, FLOT1, ICAM1, EpCam, ANXA5, and TSG101, and a Golgi-matrix marker of cell contamination, GM130. The protocol was undertaken following the manufacturer’s instructions using 93 μg of unlysed EV protein from pooled fractions 8–10, quantified using a ‘Pierce™ BCA Protein Assay Kit’ (Thermo Fisher Scientific). This equated to 10 μL of lysed concentrated EV of 5 pooled *M. bovis* positive serum samples and 22 μL of concentrated EV of 5 pooled *M. bovis* negative serum samples. Membranes were imaged as above.

### 2.5 Protein extraction

Concentrated EVs were defrosted at room temperature and lysed in 200 μL of a freshly prepared lysis buffer (7 M urea, 2 M thiourea, 1% sodium deoxycholate (SDC) in 100 mM ammonium bicarbonate (AB) buffer), heated at 95 °C for 10 min, and sonicated three times for 5 s each with a VC-50 Vibra Cell Ultrasonic Processor, 120 V, 50 W (Sonics & Materials Inc., Newtown, CT, USA) at an output of 30. Samples were kept on ice between each sonication and, once sonication was completed, centrifuged at 12,000×*g* for 10 min at 4 °C. Proteins were precipitated using methanol/chloroform precipitation (28), resuspended in 25 μL of 100 mM ammonium bicarbonate buffer, and vortexed thoroughly to dissolve the pellet. The protein concentration was measured using a UV–Vis Spectrophotometer (Implen NanoPhotometer), and 14 μg of protein transferred to a new protein LoBind tube (Eppendorf) and topped up to 50 μL with 0.1 M ammonium bicarbonate buffer. The samples were then reduced with 1.25 μL of 200 mM dithiothreitol (DTT) at 56 °C for 45 min in a ThermoMixer (Eppendorf), followed by alkylation by the addition of 4 μL of 200 mM iodoacetamide at room temperature in the dark for 30 min. Proteins were digested into peptides with 1 μg of trypsin/Lys-C mix (1:25 ratio) and incubated overnight in a thermomixer at 37 °C. The peptide mixture was desalted using Pierce™ 100 μL C18 Tips (Thermo Fisher Scientific), according to manufacturer’s instructions, dried in a vacuum concentrator, and stored at −20 °C until analysis. The dried digests were resuspended in 50 μL 0.1% formic acid prior to injection on the mass spectrometer.

### 2.6 Liquid chromatography with trapped ion mobility spectrometry and tandem mass spectrometry (LC-TIMS-MS/MS)

The LC-TIMS-MS/MS data acquisition was undertaken using a nanoElute nanoflow ultrahigh pressure LC system (Bruker Daltonics, Bremen, Germany) coupled to the timsTOF Pro2 mass spectrometer (Bruker Daltonics) operating in positive ion mode, using a CaptiveSpray nanoelectrospray ion source (Bruker Daltonics). The source-drying temperature was set to 180 °C with a gas flow of 3 L/min and capillary voltage at 1,500 V.

For each of the samples, 200 ng of peptides was loaded on a reversed-phase column (25 cm length, 75 μm inner diameter, 1.6 μm particle size, and 120 Å pore size) with a pulled emitter tip (IonOpticks, VIC, Australia). Peptides were separated at 50 °C at a flow rate of 300 nL/min using a linear gradient of mobile phase A (0.1% formic acid in water) and mobile phase B (0.1% formic acid in 100% acetonitrile) from 2 to 35% B in 60 min followed by a steep increase to 95% B in 10 min. The column was held at 95% B for 5 min, returned to 2% B over 2 min, and then re-equilibrated at 2% B for 13 min resulting in a total runtime of 90 min.

The timsTOF Pro 2 was operated in data-independent acquisition parallel accumulation–serial fragmentation (diaPASEF) mode. A polygon filter was applied to exclude low m/z, singly charged ions from PASEF precursor selection. The TIMS-MS survey scan was acquired between 0.6–1.6 V s/cm^2^ and 100–1,700 m/z with a ramp time of 166 ms and an acquisition ramp rate of 9.43 Hz. diaPASEF scans were acquired across 32 defined 25 Th isolation windows from 400 to 1,200 m/z and a total estimated diaPASEF cycle time of 1.8 s.

### 2.7 Protein identification and label-free quantification

The diaPASEF files were analyzed using a library-free direct data independent acquisition (DIA) + (Deep) approach in Spectronaut (version 19.3, Biognosys, Switzerland). For the database search using the Pulsar search engine, the following settings were used: a SwissProt *Bos taurus* database, combined with *Mycoplasma bovis* TS01 sequences (downloaded on 26 October 2022, containing 10,558 sequences), Trypsin/P and LysC as enzymes, specific digest type, with up to two missed cleavages allowed; peptide length ranged from 7 to 52 amino acids; carbamidomethyl of cysteine as fixed modification, carbamidomethyl (N-term), carbamyl (N-term), carbamylation (KR), deamidation (NQ), and oxidation (M) as variable modifications, and 1% false discovery rate (FDR) for peptide-spectrum matches (PSM), peptide, and protein group identification. Mass tolerance of MS and MS/MS was set as dynamic with a correction factor of one.

### 2.8 Statistics

For DIA analyses, the following settings were applied: Source specific index retention time calibration with a local (non-linear) RT regression was applied. The precursor and protein cutoffs were kept as default. The proteotypicity filter was set to ‘only protein group specific’. A cross-run global normalization strategy was followed by normalizing on median, and the MaxLFQ method was used for protein quantification. The quantity was determined at the MS2 level using the area. The diaPASEF dataset was analyzed with a sparse *q*-value, and no imputation was performed. Protein inference was undertaken via ID-Picker. Post-analysis, differential abundance testing was undertaken using an unpaired *t*-test. Gene ontology annotations were performed using the *Bos taurus* EBI file (GO annotations EBI, containing 20,198 entries).

### 2.9 Data visualization

Data visualization was undertaken using R (version 3.6.1) and the tidyverse set of data transformation tools and the ggplot package. Pathway analysis was performed using PANTHER, and protein–protein interactions were assessed using STRING. The exocarta database was used to compare protein identifications with known exosomal proteins.

## 3 Results

### 3.1 Characterization of extracellular vesicles

**Figure 1:**
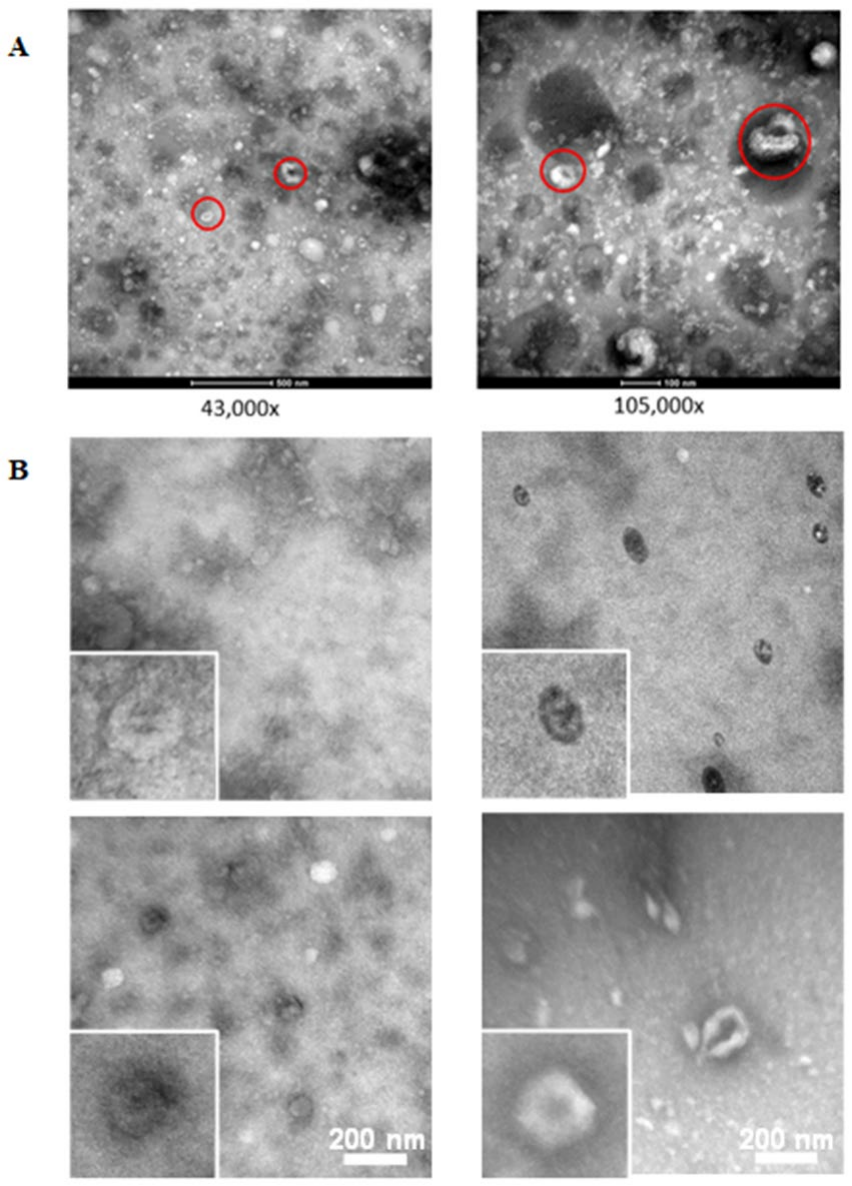
Transmission electron microscopy of small extracellular vesicles isolated using size exclusion chromatography (iZON). **(A)** Vesicles isolated from a representative negative pool at 43,000× and 105,000× magnification. EVs demonstrating the characteristic ‘donut’ shape after fixation and negative staining are circled in red. **(B)** Vesicles from *Mycoplasma bovis* negative (left) and positive (right) cows at 105,000× magnification and 135,000× magnification in insert.

Images using TEM confirmed the heterogeneity and integrity of EV from blood of *M. bovis* negative and *M. bovis* positive cows demonstrating populations of small and large vesicles resembling exosomes (30–150 nm) and microvesicles (> 150 nm) with characteristic cup-shaped morphology typical of exosomes (Figure 1). It is likely that indiscriminate electron-dense particles were highly abundant serum proteins as these are co-purified during the isolation of sEV.

**Figure 2:**
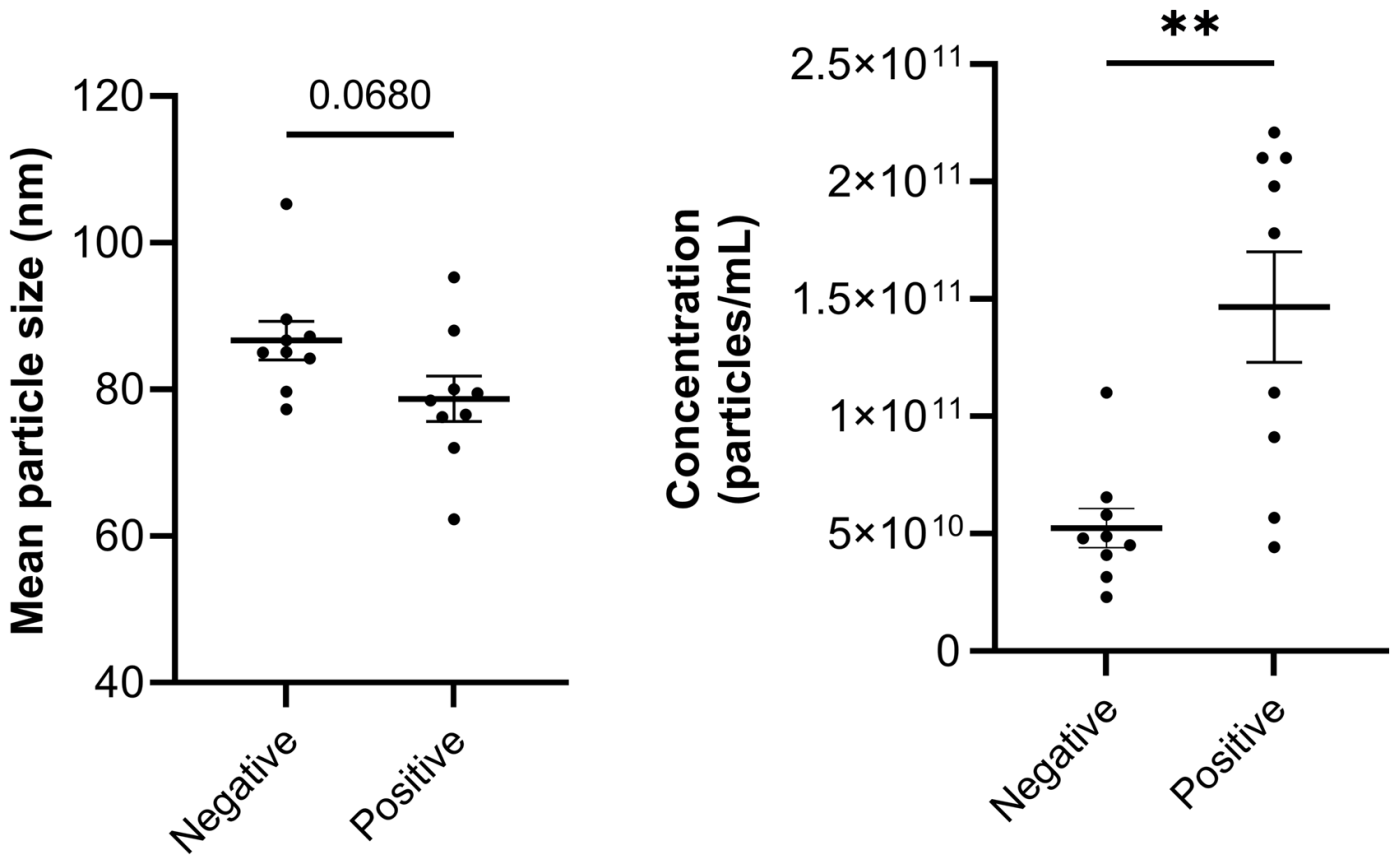
Size and concentration of the small extracellular vesicles (sEV) isolated from pooled serum of cows that were negative or positive for *Mycoplasma bovis* infection. Small EV were isolated using qEV10 35 nm second generation size exclusion chromatography (iZON) columns and measured using nanoparticle tracking analysis.

The size of isolated sEV measured using NTA indicated a mean particle size across all samples of 83 nm ± 9.3 nm (mean ± SEM). There was a significant (*p* < 0.01) difference of sEV particle concentration between negative and positive samples with more sEV isolated from serum positive for *M. bovis* infection. The mean particle concentration was 5.2 × 10^10^ ± 8.36 × 10^9^ particles/mL (mean ± SEM) in negative samples and 1.5 × 10^11^ ± 2.36 × 10^10^ particles/mL of sEV isolated from the positive serum (Figure 2). There tended (*p* = 0.07) to be smaller particles in positive samples than negative samples; size of sEV isolated from negative serum samples was 87 nm ± 2.6 nm (mean ± SEM) and 79 ± 3.1 nm (mean ± SEM) from positive serum samples (Figure 2). There was no significant difference in the volume of serum used for sEV isolation from the pooled samples (*p* = 0.35).

**Figure 3:**
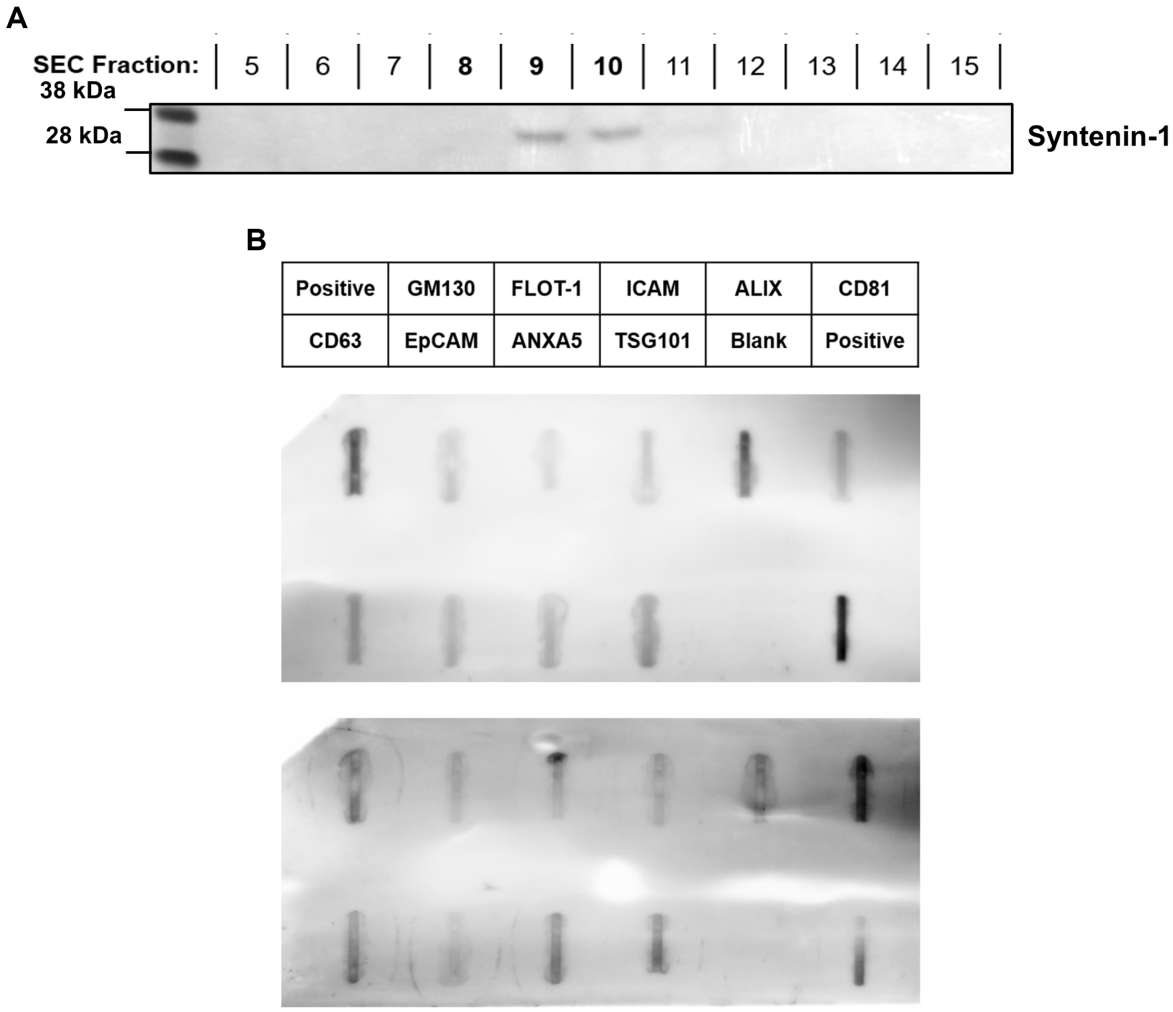
Characterization of extracellular vesicles using Western blot **(A)** and Exo-Check™ Exosome Antibody Array **(B)**; (i) = representative fractions collected from *M. bovis* positive serum sEV isolation demonstrating syntenin-1 expression and (ii) = representative sample from *M. bovis* negative serum sEV isolation.

Exosomal protein markers were identified in sEV-containing fractions. Of the proteins measured using Western blot analysis of fractions 5–15 from a representative single serum sample, we identified the presence of syntenin-1 in fractions 9 and 10 (Figure 3A). Investigation of EV markers in representative *M. bovis* positive and *M. bovis* negative pooled samples using Exo-Check™ arrays (Figure 3B) indicated the presence of CD63, CD81, ALIX, FLOT1, ICAM1, EpCam, ANXA5, and TSG101.

### 3.2 Proteomic analysis of extracellular vesicles

Label-free quantitative proteomics profiling was used to determine whether differences in protein abundance could be observed between the pooled *M. bovis* positive and *M. bovis* negative EV samples. As purified serum sEV samples are still known to contain high-abundant blood-derived proteins (29), such as fibronectin, we undertook the analyses on a state-of-the-art TIMS-Q-TOF instrument, which enables us to apply 4D proteomic analyses in combination with data-independent analysis to maximize the number of identified and relatively quantified high-quality peptide sequences and thus protein identifications.

**Figure 4:**
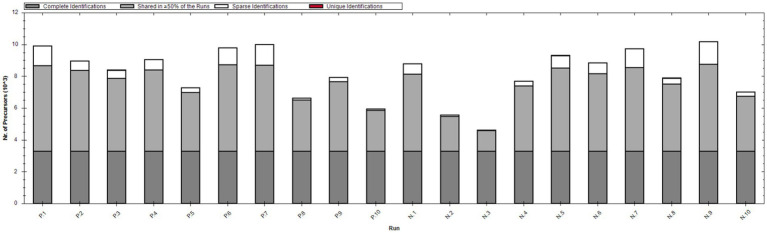
Overview of the number of protein identifications in small extracellular vesicles in pooled serum samples from dairy cows that were positive (P) and negative (N) for *Mycoplasma bovis* infection. Color coding on the bars indicates the number of proteins identified in all samples (complete identifications; dark gray), proteins that were identified in >50% of the runs (shared in >50% of the runs; light gray), and proteins identified in >20% of the samples (sparse identifications; white).

Across all samples, 10,296 peptides were identified. After quality control filtering (FDR < 1% at the protein level, minimum two unique peptides per protein), 695 non-redundant proteins were confidently identified and quantified. Two group pools (N2 and N3) had fewer protein identifications than other pools (Figure 4), potentially reflecting lower EV yield or sample-specific variance.

### 3.3 Differences in protein profiles

**Figure 5:**
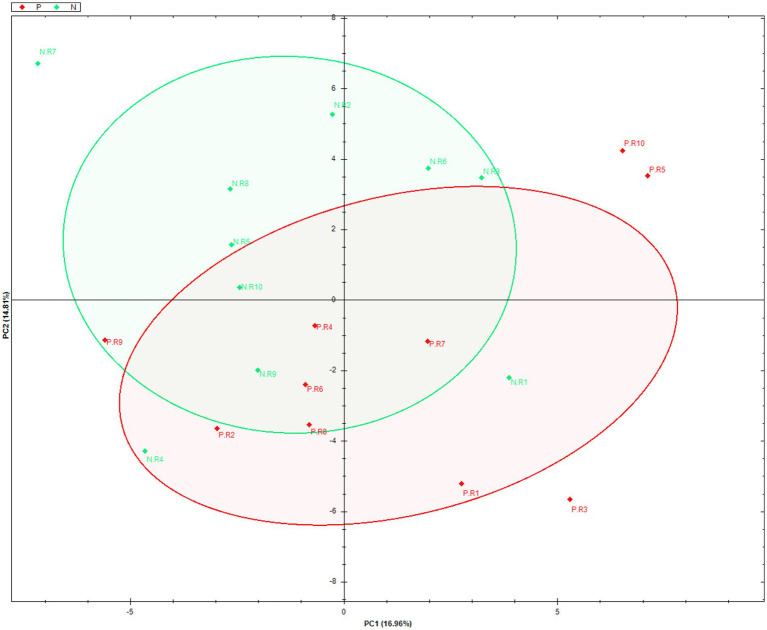
Principal component analysis (PCA) of protein profiles identified and relatively quantified in small extracellular vesicles from serum of dairy cows positive (P; red) compared with negative (N; green) for *Mycoplasma bovis* infection.

To investigate differences in protein profiles between all the samples, a multivariate data analysis approach was performed using a principal component analysis (PCA). In Figure 5, a comparison of the profiles in the first two principal components which explain the largest variance in the data is visualized.

The two principal components only explained 31.75% (PC1 + PC2 variance) of the variance, with no clear discrimination between the positive and negative groups observed. This indicated a large variation among samples and was confirmed by coefficient of variance (CV) analyses; the pooled positive samples had a median CV of 55.3%, and the pooled negative samples had a median CV of 62.5%.

### 3.4 Differential protein abundance

**Figure 6:**
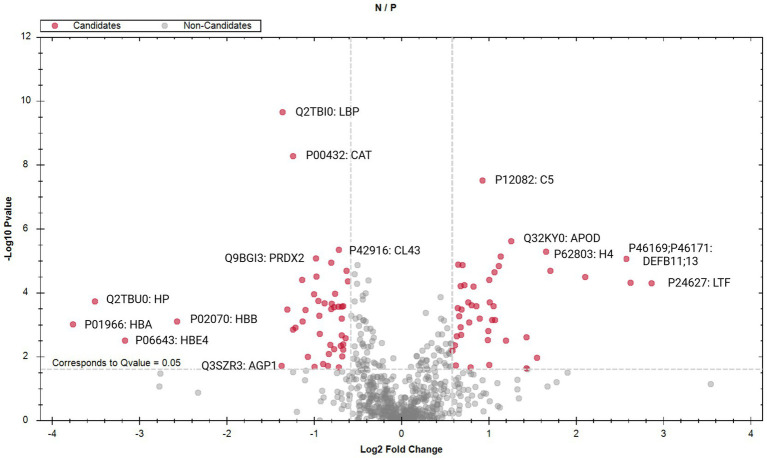
Volcano plot to identify small extracellular vesicle proteins that are significantly different (*q* < 0.05; log_2_ fold change >1) between animals positive for *Mycoplasma bovis* infection (P) compared with animals that are negative for infection (N). Red data points indicate proteins significant with infection status (candidates; *q* < 0.05), gray data points indicate *q* > 0.05 (non-candidates). A total of 15 significant differentially abundant proteins between cows of differing infection status (P vs. N) have been annotated with BioRender: Ross, M. (2026); https://BioRender.com/iu6j52e.

Differential abundance analysis indicated there were 90 proteins that were significantly (*q*-value < 0.05) different in sEV of infected animals compared with controls (Supplementary Table 2). Abundance of sEV-associated proteins CD9, CD63, CD81, and HSP90 (*α* and *β*) was identified in our dataset and was not significantly different between infection status groups (Supplementary Figure 1; *p* > 0.05). A volcano plot (Figure 6) indicates significant proteins between *M. bovis* negative (not-infected) and *M. bovis* positive (infected) samples in red using parameters *q*-value < 0.05 and a log_2_ fold change > 1.

**Figure 7:**
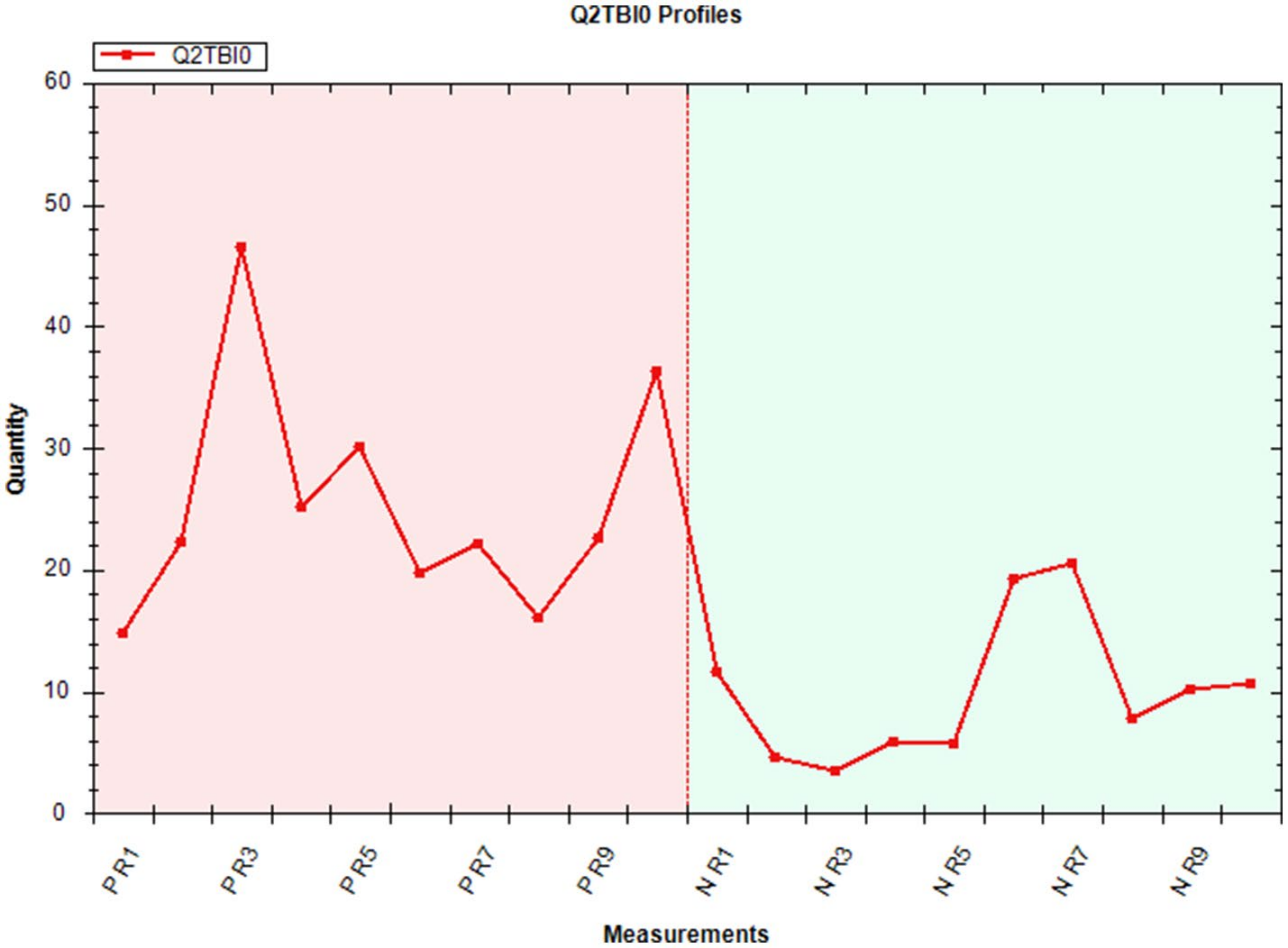
Abundance plot of lipopolysaccharide-binding protein (Q2TB10) in small extracellular vesicles from serum of *Mycoplasma bovis* positive (P; red) and negative (N; green) dairy cows.

The 20 proteins that are most significantly differentially abundant between sEV of the two infection status groups (negative vs. positive) are presented in Table 1. The Log_2_ ratio demonstrates abundance of these proteins in negative/positive infection status groups, i.e., lipopolysaccharide-binding protein (LBP), catalase, collectin 43, peroxiredoxin 2, spleen trypsin inhibitor, platelet glycoprotein 4, angiopoietin, and ferritin light chain are all significantly (*q* < 0.05) more abundant in sEV from positively infected animals. To demonstrate abundance and the variability in sEV pools, we have presented the abundance profile of an example protein, LBP, in negative (green) and positive (red) pools (Figure 7).

**Table 1:** Most significant proteins.

Protein ID(s)	Log_2_ ratio	Protein name	*q-*value
Q2TBI0	−1.36	Lipopolysaccharide-binding protein	8.18E−08
P00432	−1.24	Catalase	9.62E−07
O46415	−1.14	Ferritin light chain	6.54E−04
Q9BGI3	−0.98	Peroxiredoxin-2	3.50E−04
O18920	−0.97	Angiopoietin-1	5.90E−04
P04815	−0.8	Spleen trypsin inhibitor I	3.81E−04
P42916	−0.72	Collectin-43	3.17E−04
P26201	−0.63	Platelet glycoprotein 4	4.77E−04
Q0VCM5	0.65	Inter-alpha-trypsin inhibitor heavy chain H1	3.81E−04
F1N2K1	0.7	Prenylcysteine oxidase 1	3.81E−04
P12082	0.93	Complement C5a anaphylatoxin	3.68E−06
P12344	1.01	Aspartate aminotransferase, mitochondrial	6.54E−04
P80109	1.07	Phosphatidylinositol-glycan-specific phospholipase D	4.88E−04
P22226	1.11	Cathelicidin-1	3.81E−04
Q3T0E5	1.14	Adipocyte plasma membrane-associated protein	3.50E−04
Q32KY0	1.26	Apolipoprotein D	2.21E−04
P62803	1.66	Histone H4	3.17E−04
P17690	1.71	Beta-2-glycoprotein 1	4.77E−04
O18815	2.1	Beta-defensin C7 (Fragment)	5.98E−04
P46169; P46171	2.57	Beta-defensin 1; Beta-defensin 13	3.50E−04

The 20 small extracellular vesicle proteins that demonstrate the greatest significance (smallest *q*-value) in differential abundance between dairy cows negative (N) or positive (P) for *Mycoplasma bovis* infection. Log_2_ ratio was calculated by protein abundance in N/P; a negative Log_2_ ratio demonstrates lower protein abundance in N compared with P samples.

The 20 significant (*q* < 0.05) proteins that demonstrated the most differential abundance between serum sEV with positive infection status than negative infection status are presented in Table 2. As above, proteins with a log_2_ ratio <1 indicate they are significantly higher in abundance in positive samples than negative samples and, conversely, positive log_2_ ratios (>1) demonstrate proteins in greater abundance in the negative group than the positive group. For example, hemoglobin subunits *α* and *ε* and haptoglobin are the three proteins that have the lowest average abundance in negative pools than positive pools. It should be noted, however, that these results are driven by very high abundance in two positively infected pools (P5 and P10).

**Table 2:** Most differentially abundant proteins.

Protein ID(s)	Log_2_ ratio	Protein name	*q*-value
P01966	−3.76	Hemoglobin subunit alpha	4.81E−03
Q2TBU0	−3.51	Haptoglobin	1.75E−03
P06643	−3.17	Hemoglobin subunit epsilon-4	1.16E−02
P02070	−2.57	Hemoglobin subunit beta	4.00E−03
Q3SZR3	−1.37	Alpha-1-acid glycoprotein	4.32E−02
Q2TBI0	−1.36	Lipopolysaccharide-binding protein	8.18E−08
P02662	−1.31	Alpha-S1-casein	2.25E−03
P00432	−1.24	Catalase	9.62E−07
A4IFH5	−1.24	Alanine aminotransferase 1	6.36E−03
Q2KJ51	−1.21	Angiopoietin-related protein 4	5.77E−03
Q32KY0	1.26	Apolipoprotein D	2.21E−04
Q3ZBD7	1.43	Glucose-6-phosphate isomerase	9.75E−03
P81623	1.43	Endoplasmic reticulum resident protein 29	4.80E−02
A1L595	1.56	Keratin, type I cytoskeletal 17	2.89E−02
P62803	1.66	Histone H4	3.17E−04
P17690	1.71	Beta-2-glycoprotein 1	4.77E−04
O18815	2.1	Beta-defensin C7 (Fragment)	5.98E−04
P46169; P46171	2.57	Beta-defensin 11; Beta-defensin 13	3.50E−04
P68432; P84227; Q5E9F8	2.62	Histone H3.1; Histone H3.2; Histone H3.3	7.06E−04
P24627	2.87	Lactotransferrin	7.06E−04

The top 20 significant (*q*-value < 0.05) small extracellular vesicle proteins with the greatest difference in abundance (|Log_2_ ratio|) between dairy cows negative (N) or positive (P) for *Mycoplasma bovis* infection. Log_2_ ratio was calculated by protein abundance in N/P; a negative Log_2_ ratio demonstrates lower protein abundance in N compared with P samples.

### 3.5 Interaction networks and pathway analysis

**Figure 8:**
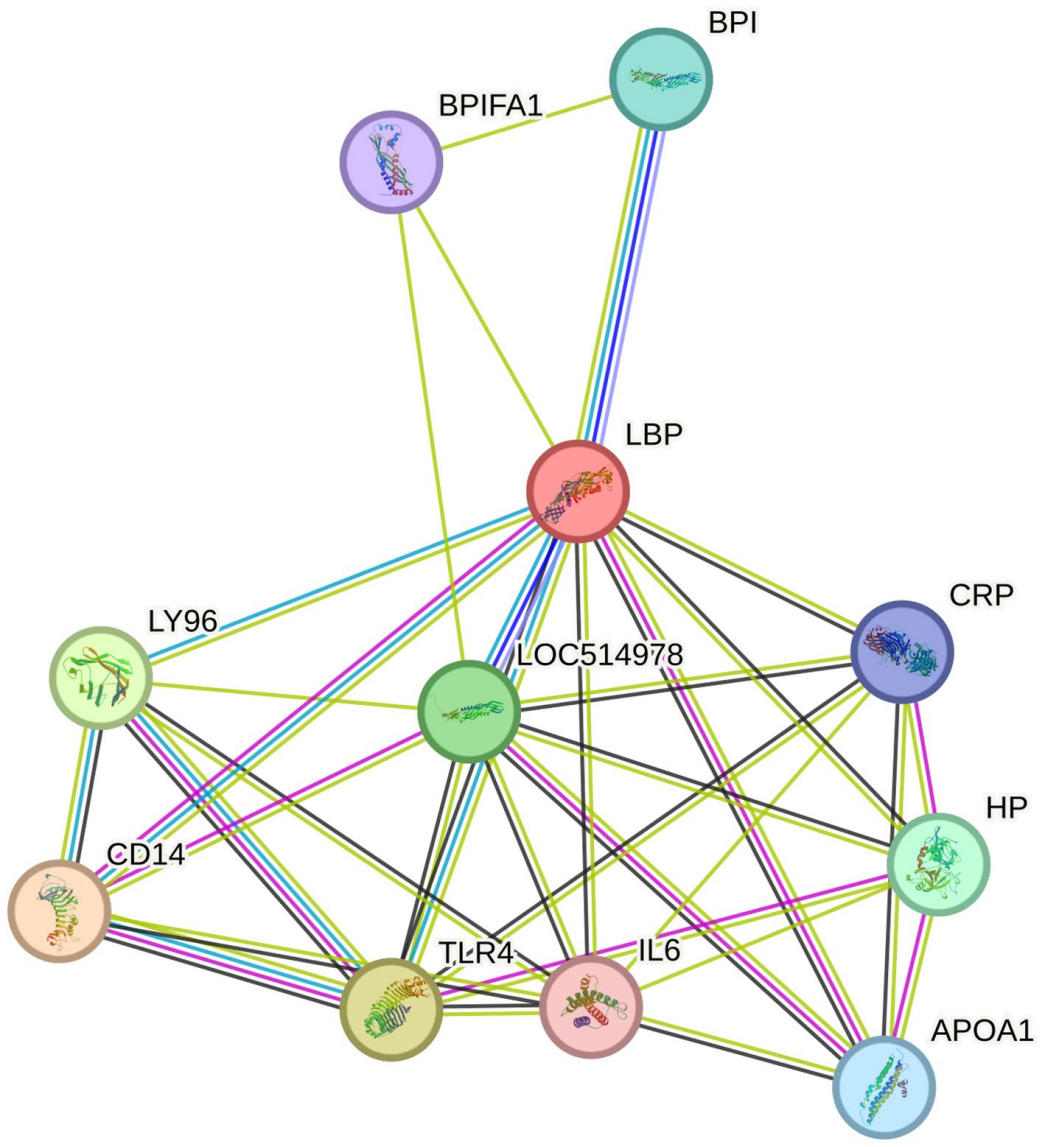
STRING pathway analysis of protein interactions with lipopolysaccharide-binding protein (LBP).

Protein–protein interaction networks of LBP using STRING analysis indicated functional protein interactions that were among the DEP including haptoglobin (HP) and CD14 (Figure 8).

**Figure 9:**
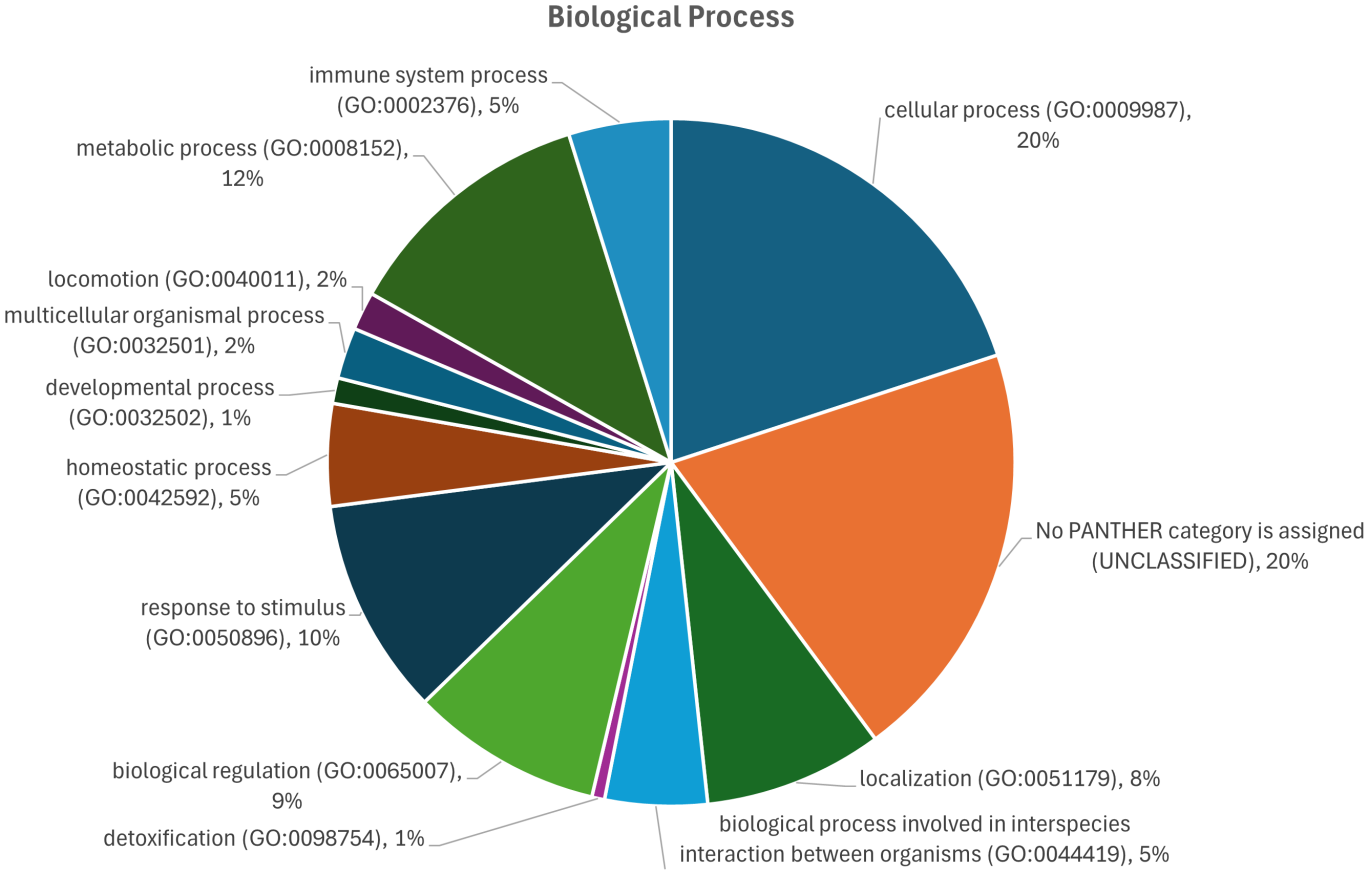
Biological process classification according to gene ontology (GO) terms of the significantly (*q* < 0.05) differentially abundant proteins in sEV from cows of different infection statuses.

Functional pathway analysis of the host (*Bos taurus*) response proteome using classification on biological process gene ontology (GO) terms of the significantly (*q* < 0.05) differentially abundant sEV proteins indicated that, of those that could be classified, the largest group of proteins are involved in cellular processes (20%; Figure 9). Additionally, proteins were representative of metabolic processes (12%), response to stimulus (10%), biological regulation (9%), immune system process (5%), and interaction between organisms (5%).

## 4 Discussion

This study characterized the serum sEV proteome of dairy cows naturally infected with *M. bovis* compared with uninfected controls. We identified 90 proteins with significantly different abundances between groups, including immunomodulatory acute phase proteins (APPs), complement pathway components, oxidative stress enzymes, antimicrobial peptides, and histones. The proteomic profile of sEV from infected cows was consistent with an inflammatory and pathogen-response state, sharing similarities with sEV signatures in other bacterial infections in ruminants and humans as reported below.

Small EV cargo of *M. bovis* infected animals indicates an increased inflammatory state relative to healthy animals. Inflammation markers LBP, HP, and alpha-1 acid glycoprotein (α1AGP) are APP that have greater abundance in sEV from *M. bovis* positive animals than *M. bovis* negative animals. LBP has been demonstrated to increase systemically following intramammary challenge with *M. bovis* (30). Furthermore, α1AGP and HP are known to increase in blood during mastitis infection and are positively associated in dairy cows during post-partum inflammation (31, 32); in addition, α1AGP inhibits Mycoplasma (*M. pneumoniae*) adherence to host cells, suggesting a protective role by mediating bacterial attachment (33). In context of sEV protein cargo biomarkers, LBP, HP, and serum amyloid A1 (another positive APP) were all increased in circulating exosomes of patients infected with *Mycobacterium tuberculosis* (34, 35), and in serum of *M. bovis*-infected cattle, HP and SAA are upregulated (36). It should be noted that there is large variation in the relative abundance of APP across our samples, likely owing to variation in sampling time points and farms, indicating that abundance will vary across infected individuals. For example, the significant difference in abundance of HP is driven by high expression in two positive samples and, as pooled serum was used in our study, we cannot discriminate the variation between individuals. Therefore, these results should be interpreted with caution and characterized further. Overall, the results indicate that sEV protein cargo during subclinical *M. bovis* infection is consistent with increased inflammation that is comparable to other inflammatory conditions and bacterial infections in dairy cows and humans.

Protein–protein interaction analysis of APP highlights their immunomodulatory function, which can exert both pro- and anti-inflammatory effects suggesting context-dependent changes to the acute phase response during *M. bovis* infection. Proteins that interact with LBP from STRING analysis demonstrate that functional partners are differentially abundant between *M. bovis* positive and *M. bovis* negative samples. HP is a functional partner and has an important role in inflammation as an APP but also exhibits anti-inflammatory action by reducing oxidative and inflammatory damage by binding and clearing hemoglobin (37). Hemoglobin is associated with sEV from erythrocytes under non-infectious but pathological conditions (kidney disease) (38, 39). Hemoglobin subunits are among our differentially abundant proteins and driven by the same samples, suggesting there is increased hemolysis in some infected animals that could require clearance by HP/hemoglobin complexes (40). Further support for altered iron control during *M. bovis* infection in our dataset is lower abundance of transferrin (serotransferrin and lactotransferrin) in EV cargo of *M. bovis* positive cows. Transferrin and lactotransferrin are iron-binding glycoproteins, which can disrupt microbial growth and pathogenesis (41, 42). Therefore, alteration of these systems may facilitate immunomodulation mediated by the acute phase response during *M. bovis* infection.

Further evidence of APP-mediated immunomodulation during *M. bovis* infection is apolipoproteins as binding partners. For example, APOA1 is demonstrated to be a binding partner of LBP, while it is not differentially abundant between infection status groups, other members of the apolipoprotein family are, including, APOA2, APOA4, APOD, and APOE. Another differentially abundant protein in this pathway is CD14, which is greater in abundance in sEV from *M. bovis* positive cows than *M. bovis* negative cows. CD14 is a monocyte-associated receptor involved in the innate immune response during inflammation (43). Infection with *M. bovis* has been demonstrated to associate with monocytes/macrophages and interfere with function by delaying apoptosis (44, 45). Furthermore, sEV derived from macrophages infected with *M. pneumoniae* induce the production of inflammatory cytokines by uninfected macrophages *in vitro*, providing evidence of downstream function of sEV on the immune system during Mycoplasma infection (46). These results indicate the activation of LBP-associated inflammation during *M. bovis* infection, which can be distinguished in sEV cargo of infected animals, and functional analysis of *M. bovis*-derived sEV during natural infection may elucidate successive immune pathways that are upregulated.

Differential abundance of complement-related proteins indicates immunomodulation of classical and lectin pathways during *M. bovis* infection. Collectin-11 and -43, both more abundant in sEV from infected cows, are pattern-recognition molecules that can trigger complement activation at epithelial surfaces (47, 48). Furthermore, complement C1q subunits A and B and C1s serine protease were also enriched in infected sEV and C5a demonstrated lower abundance, possibly reflecting downstream regulation or consumption. Given that complement proteins are often glycosylated and that altered glycosylation has been demonstrated in mycobacterial infection (49, 50), targeted glycoproteomic profiling could elucidate their functional state in *M. bovis* infection.

Differential abundance of immune response-related proteins may indicate a downregulation of the innate immune response in *M. bovis* positive animals. There is lower abundance of proteins involved in the innate immune response of epithelial cells, such as ICAM3, ITGB2, and beta-defensins (DEFB6, C7, B11, and B13), in *M. bovis* positive animals than *M. bovis* negative animals. Integrin ITGB2 is part of the receptor for ICAM3, involved in leukocyte adhesion and neutrophil migration (51). Beta-defensins are antimicrobial peptides produced at the site of infection and are well-characterized for their immunomodulatory activity (52). Beta-defensins have been demonstrated to be downregulated during infection compared with uninfected controls. For example, beta-defensin 1 is downregulated in response to in both *in vitro* and *in vivo* murine models of *Cryptosporidium parvum* infection (53). Furthermore, beta-defensin genes, including those encoding DEFB6 and B7, are downregulated in nasopharyngeal cells of SARS-CoV-2 infected patients compared with control patients (54). In dairy cows, expression of the gene encoding DEFB6 and DEFB7 in neutrophils has been demonstrated to be downregulated in post-partum disease including retained placenta and metritis (55). We demonstrate that beta-defensins likely have a role in the context of *M. bovis* infection in dairy cows that should be investigated further.

Among our dataset, we have identified differentially abundant proteins that have demonstrated promise as biomarkers of a diseased state, but our results are inconsistent. In our dataset, cathelicidin-1 and cathelicidin-4 are differentially abundant between *M. bovis* infection status groups with lower abundance in sEV from *M. bovis* positive cows than *M. bovis* negative cows, which corresponds with defensin abundance. Previous research demonstrates that sEV-associated cathelicidin-3 has been identified as an early diagnostic biomarker of devil facial tumor disease in Tasmanian devils (56). Similar to defensins, cathelicidins are a family of peptides with antimicrobial activity (57). Previous research in cows indicates that cathelicidin is a candidate biomarker of clinical mastitis, with greater abundance in milk from mastitic quarters compared with healthy controls (58). This would suggest that cathelicidins likely play an important role in bovine defense against bacterial infection; however, we have not identified cathelicidins as a candidate biomarker for *M. bovis* due to lower abundance in EV of infected animals.

Proteins with greater abundance in sEV from *M. bovis* positive cows indicate these animals are experiencing oxidative stress during *M. bovis* infection. Enzymes peroxiredoxin 2, catalase, and glutathione synthetase are all more abundant in sEV of *M. bovis* positive cows than *M. bovis* negative cows. Catalase, peroxiredoxin 2, and glutathione peroxidase are the main antioxidant enzymes of erythrocytes to prevent damage from reactive oxygen species (ROS) (59). While glutathione peroxidase is not differentially abundant in our dataset, glutathione synthetase is, which is the enzyme that produces glutathione, a co-factor for glutathione peroxidase (60). These enzymes work to enzymatically reduce hydrogen peroxide, thereby decreasing oxidative stress in erythrocytes (61). During infection with *M. bovis*, there is an increase in ROS, as demonstrated *in vitro* following bacterial inoculation (62). Furthermore, these results complement the data discussed above regarding oxidative stress and the acute phase response. Addressing oxidative stress in the context of *M. bovis* infection may be a potential avenue for disease management (63). In the context of diagnostics, the proteins identified in sEV of *M. bovis* positive cows align with greater oxidative stress in these animals compared with healthy counterparts, which may have a role in bacterial pathogenicity.

The lower abundance of multiple histone isoforms (H3.1, H3.2, H3.3, and H4) in sEV from infected cows mirrors the findings from *in vitro M. bovis* infection models. The results presented here further align with *M. bovis* infection *in vitro*, whereby proteins with lower abundance in sEV from *M. bovis* positive cows than *M. bovis* negative cows include histone proteins (histone H3.1, 3.2, 3.3, and histone H4). This aligns with an *in vitro* model of *M. bovis* infection demonstrating that histones have lower abundance in sEV of cells co-cultured with *M. bovis* compared with cells alone (64). The biological role of histones in sEV remains unresolved, but they may participate in nucleic acid packaging and intercellular communication to maintain DNA integrity (65–67). It has been established that negatively charged DNA interacts with positively charged histone proteins within sEV, forming complexes that can influence sEV structure and function in cancer patients (68). Reduced histone content could, therefore, reflect altered chromatin dynamics in infected immune cells, as has been observed in other intracellular bacterial infections (69, 70). The reduction of histones in sEV may indicate that *M. bovis* infection leads to changes in chromatin accessibility within host immune cells, decreasing the formation of nucleosome-like complexes, and limiting the exchange of DNA-based biomarkers through sEV. Consequently, this could impair immune regulation and host responses to infection. This hypothesis warrants direct validation, ideally in unpooled samples with parallel transcriptomic profiling.

Further evidence that our results are consistent with a diseased state in *M. bovis* positive animals is the differences in exosome number and size between infection status groups. There is greater concentration of sEV, and the sEV size tends to be smaller in *M. bovis* infected animals compared with healthy animals. This aligns with previous research that indicates more sEV are apparent in blood and there are changes in EV biogenesis during bacterial infection compared with healthy states, which alters EV populations (71). During infection by *Mycoplasma* sp., sEV can be derived from bacterial cells or from infected host cells and, under stress conditions, the number of sEV increases and the size of sEV decreases (72, 73), which aligns with results demonstrated in the current study. While not diagnostic, our results suggest a shift toward a diseased state consistent with bacterial infections in *M. bovis*-positive cows.

## 5 Conclusion

This study delivers the first in-depth analysis of the serum sEV proteome in dairy cows naturally infected with *M. bovis*, unveiling an inflammatory and antimicrobial signature. There is evidence of an immunomodulatory function of sEV during infection with involvement of acute phase proteins, complement pathway factors, and oxidative stress enzymes, along with potential roles of beta-defensins and cathelicidins in host defense. While no single protein emerged as a definitive diagnostic biomarker, the suite of differentially abundant proteins, along with infection-related changes in vesicle number, size, and histone content, provides new molecular insights into host–pathogen interactions and EV biogenesis during bacterial infection. These findings lay the groundwork for subsequent validation of vesicle-associated protein candidates as diagnostic panels and underscore the broader utility of sEV proteomics for unravelling complex infectious disease processes in livestock.

## Data Availability

The mass spectrometry proteomics data have been deposited to the ProteomeXchange Consortium via the PRIDE (74) partner repository with the dataset identifier PXD076220.
