# Supplementary material for: Inflammation markers are associated with small extracellular vesicle protein signatures in cows with Mycoplasma bovis
Source: Front Vet Sci. 2026 May 15;13:1787282. doi: 10.3389/fvets.2026.1787282 (PMC13218866; doi:10.3389/fvets.2026.1787282)
Supplement: Supplementary file 1 [file Image_1.pdf]

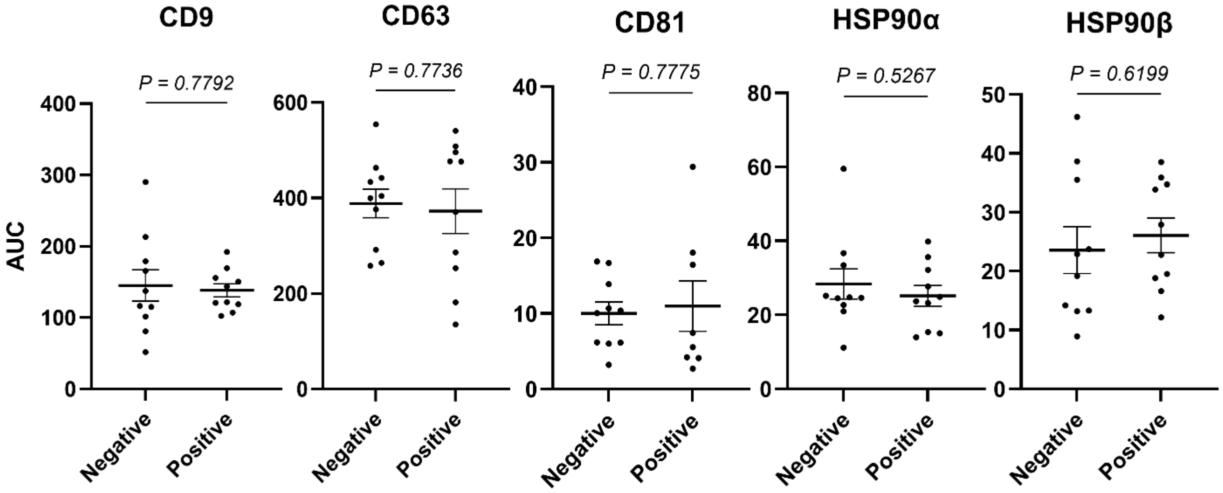

Supplementary Figure 1: Abundance of proteins associated with small extracellular vesicles. Area under the curve (**AUC**) of protein abundance determined by LC-MS/MS. Abundance of proteins in pooled samples from cows without *Mycoplasma bovis* infection (**Negative**) or with *Mycoplasma bovis* infection (**Positive**). Differential abundance analysis was undertaken using an unpaired t-test (GraphPad Prism 10.5.0).
